# Establishment of a lectotype for the species *Plesiochelys langii* Rütimeyer, 1873

**DOI:** 10.7717/peerj.1108

**Published:** 2015-08-04

**Authors:** Jérémy Anquetin

**Affiliations:** Section d’archéologie et paléontologie, Office de la culture, République et Canton du Jura, Porrentruy, Switzerland

**Keywords:** *Plesiochelys langii*, *Plesiochelys etalloni*, Plesiochelyidae, Lectotype, Nomenclature

## Abstract

**Background.** In a recent paper, we proposed a lectotype for the species *Plesiochelys langii* Rütimeyer, 1873. However, we failed to register this publication in ZooBank as required by the International Commission on Zoological Nomenclature (ICZN) for electronic publication. Although our conclusions remain unchanged, this particular nomenclatural act cannot be considered as published under ICZN regulations.

**Results.** The present work fulfills the requirements of the ICZN for a lectotype designation and has been registered in ZooBank.

## Introduction

Anquetin, Püntener & Billon-Bruyat (2014) recently reassessed the taxonomy of the Late Jurassic eucryptodiran turtles from the Swiss and French Jura Mountains. They proposed a lectotype for the species *Plesiochelys langii* Rütimeyer, 1873, but failed to register this electronic publication in ZooBank as required by the International Commission on Zoological Nomenclature (ICZN). As a result, this nomenclatural act cannot be considered as published under ICZN regulations.

The present work has been registered in ZooBank (see below) and now fulfills the requirements of the ICZN for a lectotype designation.

## Material and Methods

The electronic version of this article in Portable Document Format (PDF) will represent a published work according to the International Commission on Zoological Nomenclature (ICZN), and hence the lectotype designation contained in the electronic version is effectively published under that Code from the electronic edition alone. This published work has been registered in ZooBank, the online registration system for the ICZN. The ZooBank LSID (Life Science Identifier) can be resolved and the associated information viewed through any standard web browser by appending the LSID to the prefix “http://zoobank.org/.” The LSID for this publication is: urn:lsid:zoobank.org:pub:1F654A2A-8DBA-4574-ACD0-8FEBE393979C. The online version of this work is archived and available from the following digital repositories: PeerJ, PubMed Central and CLOCKSS.

## Systematic Paleontology

**Table:** 

**TESTUDINES Batsch, 1788**
**EUCRYPTODIRA Gaffney, 1975**
**PLESIOCHELYIDAE Baur, 1888**
***Plesiochelys langii* Rütimeyer, 1873**
1873 *Plesiochelys Langii*. Rütimeyer [new species]
1965 *Plesiochelys solodurensis*. Bräm [subjective synonymy]
2014 *Plesiochelys etalloni*. Anquetin, Deschamps & Claude [subjective synonymy]

### Taxonomic assessment

Invalid name, subjective synonym of *Plesiochelys etalloni* (Pictet & Humbert, 1857).

### Type material

NMS 123, a sub-complete carapace missing the right and posterior margins. Herein designated as lectotype (see Remarks, below). NMS 126, a shell heavily encrusted with pyritic mineralizations (paralectotype).

### Type horizon and locality

Solothurn Turtle Limestone, uppermost member of the Reuchenette Formation (Kimmeridgian, Late Jurassic), vicinity of Solothurn, Canton of Solothurn, Switzerland.

### Illustrations of type

Rütimeyer (1873: plate VI, Figs. 1 and 2); Anquetin, Püntener & Billon-Bruyat (2014: Figs. 2K and 2L).

### Remarks

The following text is mostly verbatim from Anquetin, Püntener & Billon-Bruyat (2014). Rütimeyer (1873) erected *Plesiochelys langii* based on three specimens from Solothurn, which together form the original syntype series: NMS 123, NMS 124, and NMS 126. A recent review of the available material concluded that NMS 123 and NMS 126 do not significantly differ from other specimens referred to *P. etalloni*. Therefore, *P. langii* was synonymized with *P. etalloni* (Anquetin, Deschamps & Claude, 2014). However, as previously pointed out by Bräm (Bräm, 1965), NMS 124 (the third syntype) clearly belongs to a different species and is now referred to *Thalassemys hugii* Rütimeyer, 1873, (Anquetin, Püntener & Billon-Bruyat, 2014). In order to avoid potential future issues with the taxonomic status of *P. langii*, NMS 123, the main specimen described by Rütimeyer (1873), is herein designated as the lectotype of this species.

## Institutional abbreviations

NMS: Naturmuseum Solothurn, Switzerland
